# Dynamic patterns of overexploitation in fisheries

**DOI:** 10.1016/j.ecolmodel.2017.06.009

**Published:** 2017-09-10

**Authors:** Ilaria Perissi, Ugo Bardi, Toufic El Asmar, Alessandro Lavacchi

**Affiliations:** a Consorzio Interuniversitario Nazionale per la Scienza e la Tecnologia dei Materiali (INSTM), c/o Dipartimento di Chimica, Università degli Studi di Firenze, Via della Lastruccia 3, 50019, Sesto Fiorentino, Firenze, Italy; b Dipartimento di Chimica, Università degli Studi di Firenze, Via della Lastruccia 3, 50019, Sesto Fiorentino, Firenze, Italy; c Food and Agriculture Organization, FAO, Viale delle Terme di Caracalla, 00153, Roma, Italy; d Consiglio Nazionale delle Ricerche, CNR-ICCOM, Via Madonna del Piano 10, 50019, Sesto Fiorentino, Firenze, Italy

**Keywords:** Overfishing, Lotka-Volterra, Fisheries, System dynamic

## Abstract

Understanding overfishing and regulating fishing quotas is a major global challenge for the 21st Century both in terms of providing food for humankind and to preserve the oceans’ ecosystems. However, fishing is a complex economic activity, affected not just by overfishing but also by such factors as pollution, technology, financial factors and more. For this reason, it is often difficult to state with complete certainty that overfishing is the cause of the decline of a fishery. In this study, we developed a simple dynamic model specifically designed to isolate and to study the role of depletion on production. The model is based on the well-known Lotka-Volterra model, or Prey-Predator mechanism, assuming that the fish stock and the fishing industry are coupled variables that dynamically affect each other. In the model, the fishing industry acts as the “predator” and the fish stock as the “prey”. If the model can fit historical data, in particular relative to the productive decline of specific fisheries, then we have a strong indication that the decline of the fish stock is driving the decline of the fishery production. The model doesn’t pretend to be a general description of the fishing industry in all its varied forms; however, the data reported here show that the model can describe several historical cases of fisheries whose production decreased and collapsed, indicating that the overexploitation of the fish stocks is an important factor in the decline of fisheries.

## Highlights

• For the first time, the “predator-prey” model is used for the quantitative description of an economic predatory dynamic in real-world fisheries.
• A simple “mind-sized” model highlights the driving forces that lead to the resources’ overexploitation and the collapse of a production system.
• A renewable fish stock can behave as a non-renewable resource when exploited at a speed much higher than its carrying capacity.
• Coupled feedback effects are the origin of overexploitation and collapse in real-world fisheries.

## Graphical abstract

The “mind-sized” model of overexploitation in fisheries is based on the concept that the fishing industry acts as the “predator” of the resource and that its growth and subsequent decline is directly related to the abundance of the fish stock.

**Figure:**
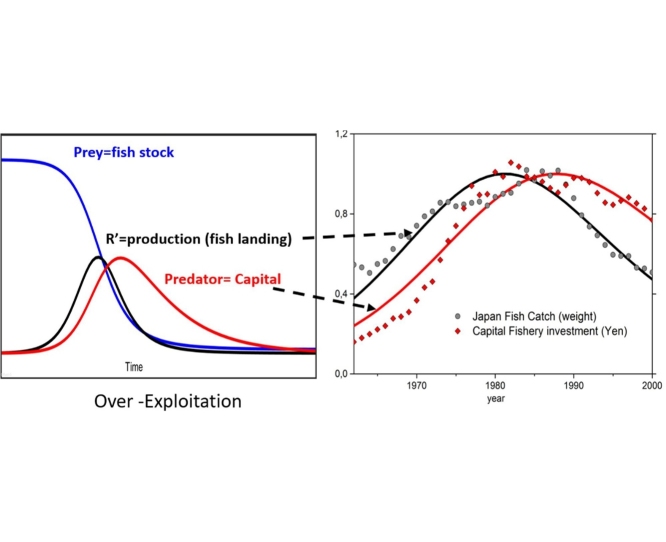

## 1 Introduction

Many of the world’s fisheries are showing a decline in the fishing yield, a phenomenon that’s clearly important for the global economy and which is often interpreted in terms of the overexploitation of the fish stock (Pauly, 2009): the fishing industry is consistently depleting the fish stock at a rate higher than the capability of the system to replenish it, as a result of biological reproduction (Lotze and Worm, 2009), (Bailey, 2016). This subject is complex and it was explored first in some early studies by Scott Gordon (Gordon, 1954) and Milner Schaefer (Schaefer, 1957). In the general field of resource overexploitation, an important influence was the work by Garrett Hardin (Hardin, 1968), known under the name of “The Tragedy of the Commons.” Hardin's model was only qualitative, but it established the patterns of overexploitation of any resource that’s exploited at a rate faster than it can reform (Roopnarine, 2013). In more recent times, fisheries have been extensively modeled, normally with considerably complex models (Schoener, 1976) and as reviewed, for instance, by Worm et al. (Worm et al., 2009).

Of course, in modeling fisheries, several factors must be taken into consideration in addition to overfishing including, e.g. the climate change (Perry et al., 2005) and that the models based on trophic chains may be over simplified (Polis and Strong, 1996). The problem, here, is to establish exactly which factors are the most important ones in generating the decline of fisheries. This is a crucial issue in the management of fisheries; overexploitation can be fought by establishing fishing quotas but we need to quantify whether there are other factors affecting yield declines. Although there appears to be a general agreement that overexploitation is an important cause of the decline of many fisheries, its extent is sometimes debated.

In the present paper, we aim at providing further evidence that overexploitation plays a central role in the collapse of at least some fisheries. We demonstrate this point by using a simple system dynamics model that takes into account the coupling of the fish stock and the fishing effort of the industry. The fact that the model can provide a good fitting with the historical data is a clear indication that the two factors influence each other in a classic feedback relationship that sees the depletion of the stock being enhanced by the effort of the industry to maintain its production rate.

The model utilized here is based on the well-known Lotka-Volterra or “prey-predator” model. Alfred Lotka (Lotka, 1925) and, independently, Vito Volterra (Volterra, 1928), (Volterra, 1931) were the first to use differential equations in order to describe the dynamics of the predator/prey interaction in biological systems and the structure of the model can be seen as an ancestor of modern “system dynamics” (Forrester, 1989). George Gause (Gause, 1932) was probably the first to seek for experimental validation of the Lotka-Volterra (LV) model and he found it, but only for very simple biological systems in the form of two species of yeasts in laboratory conditions. In general, the behavior of real biological systems turned out being too complex to be captured by the simple LV model (Hall, 1988). However, the model had been originally proposed by Vito Volterra as describing the behavior of human fisheries rather than biological systems, even though, at that time, suitable mathematical tools to fit experimental data were not available (D’Ancona, 1942). On the basis of this early idea by Volterra, we developed a simplified version of the model assuming that the fish stock behaves as a non-renewable resource when it is exploited so fast that the reproduction rate becomes a negligible parameter in the system. Some data on this approach had been reported in an earlier paper (Bardi et al., 2011) in regard to the mining industry. Even though the model we developed does not claim to be able to describe all the complex ecosystem and economics interactions that occur in a fishery, we can report several cases in which it is possible to use the model to describe the historical production patterns of fisheries. We believe that this approach can play an important role in helping people to understand the basic mechanisms of fishery management, and in particular of overexploitation. Further studies might lead to the model being usable in order to determine the carrying capacity of the system and help in the sustainable management of fisheries.

## 2 Methods

The model utilized here is based on the following couple of differential equations

*R’* *=* *−k_1_CR*

*C’* *=* *k_2_CR* − *k*_3_*C*

where “*R*” stands for the resource stock while “*C*” stands for the capital stock. The three constants of the model describe how efficiently fish is caught (*k_1_*), how efficiently the fish stock is transformed into capital (*k_2_*) and how rapidly capital is dissipated (*k_3_*). The dimensions of the constants depend on the units used for the capital and resource stocks.

This model is the same as the well-known Lotka-Volterra, predator-prey model, except in the fact that it lacks the term for the reproduction of the prey (named the resource) in the first equation.

In the original Lotka Volterra model, the prey is assumed to have an unlimited food supply and to reproduce exponentially unless subject to predation; this exponential growth is represented by a term *k_0_R* in the first equation. We will show later this term can be neglected in the study of the historical cases reported here, but see also the ‘fleet dynamic model’ reported in the work by Hilborn and Walters (Hilborn and Walters, 1992).

In the present study, the “resource stock” (or the “prey”) is the fish stock while the “capital stock” (or the “predator”) is a parameter proportional to the capital of the fishing industry in terms of vessels and other resources (including human resources). The rate of predation, *R*’, is assumed to be proportional to the abundance of both the stocks; this is represented above by −*k_1_CR*, where the efficiency of the fighting process is described by the *k_1_* coefficient.

In the second equation, *C*’ indicates the variation of the capital stock as a function of time or the ‘capital flow’. *k_2_* is a constant that describes the rate of growth of the Capital stock, also proportional to the abundance of both the fish and the capital stocks; *k_3_* is a third constant that describes the decline of the capital stock due to asset depreciation.

**Fig. 1:**
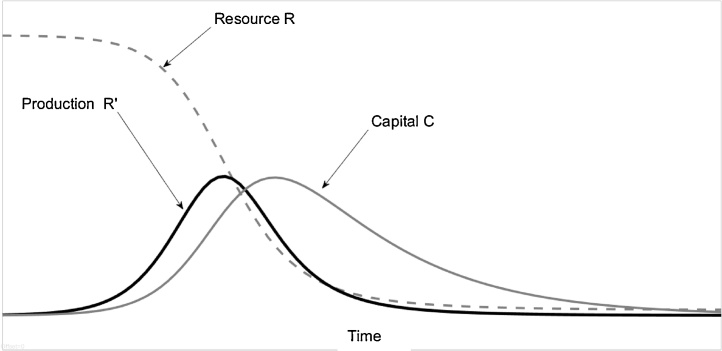
Qualitative solutions of the model. Neglecting the rate of prey reproduction imposing k_0_ = 0, the prey-predator dynamic experiences a single oscillation, showing a definitive depletion of the prey stock and the subsequent collapse of predators’ stock.

The model was implemented using MATLAB^®^ computing language and the associated Simulink toolbox. Typical results are reported in Fig. 1.

**Fig. 2:**
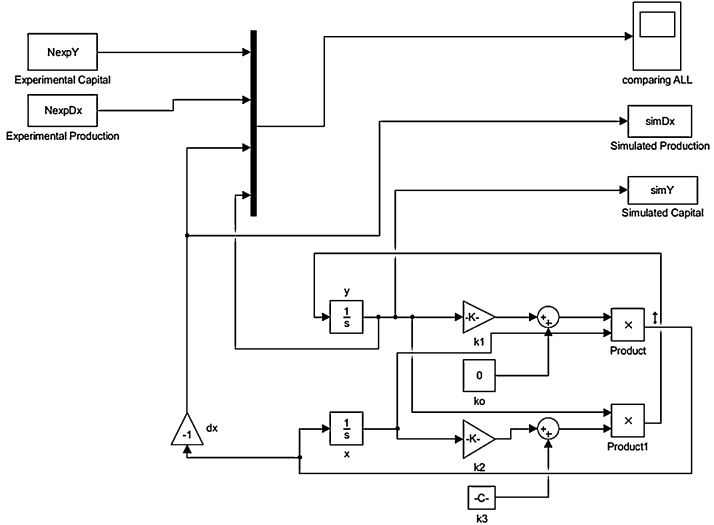
Simulink blocks’ diagram of the model. Simulink provides modeling and simulation environment for editing the Lotka Volterra equations in a graphical form. NexpDx and NexpY are, respectively, the historical fish production, R’, and the capital stocks. C; SimDx and SimY are the simulated production and capital: they correspond to the fitting curves. The Integrator block, 1/s, outputs the value of the integral of its input signal with respect to time.

In comparing the results of the model to the historical data, the three constants (*k_1_*, *k_2_* and *k_3_*) were allowed to vary until the best fitting was obtained. All the fittings reported here were generated using the unconstrained nonlinear optimization method based on the Nelder-Mead algorithm (termed “fminsearch” in Matlab). The objective is to minimize the sum of the square of the residuals (SSE, the sum of squared errors of prediction) generated by the deviations of the LV predicted data from actual empirical values of data. The fitting procedure was found to be very sensitive to the initial values of the *k_1_*, *k_2_* and *k_3_* parameters, as well as to the initial values of the stocks (*R_o_* and *C_o_*). The initial guesses for these parameters were provided by the Parameter Estimation tool available in the Simulink^®^ Design Optimization™ toolbox (Fig. 2). The Goodness of fit (GOF) was calculated by as the Normalized Mean Square Errors (NMSE) function which measures the discrepancy between the real values and the estimated ones and it was calculated by means of the Matlab Curve Fitting Toolbox. An NMSE equal to 1 represents the perfect fit, NMSE equal to zero means that the model is no better than a straight line at fitting the real data.

## 3 Results

For the present study, we collected historical data for several “non-managed” fisheries, that is for fisheries in which neither the catch nor the fishing effort was regulated in terms of quotas or other parameters. In other words, these fisheries were managed by operators with the only aim of maximizing profits. Data about the actual fish stock during the exploitation cycle are not normally available, but the historical catch data (or “landings”) – that is the flow *R*’ in the model – are commonly reported for many fisheries. A qualitative examination of these data shows that the total fishery production often shows a “bell shaped” trajectory, with production increasing until reaching a maximum and then declines.

Using the model to fit a single set of data, the landings, is possible but it does not really prove that the system is dominated by a “predator-prey” interaction.

We need to show that the two entities involved in the cycle, the predator and the prey, are linked to each other in the way described by the simplified LV model. Therefore, we need to identify also a ‘predator stock’ or, at least, some parameters that can be assumed to be proportional to the “capital stock” engaged in the fishing activity. Data on the capital investments of the fishing industry engaged in a specific fishery are not commonly available. However, “proxy” data can be used, such as, for instance, the labor force employed in the fishery industry in terms of the number of persons or in terms of their total salary. Another parameter examined was the number of fishing vessels or the tonnage of the fleet. In recent statistical surveys, also the data on the investment in currency or as measured by economic indexes of the fishing sector are available. All these parameters may be assumed to approximate the “fishing effort” represented by the *“C”* parameter in the model. In the end, the fitting procedure was developed for the two parameters that turned out to be available.

In all cases, the historical data were normalized before fitting and we found several cases where the

real world stocks, fish and capital invested in fishing, interact each other in the way described by the model.

In the following, we show results describing cases of fisheries overexploitation from the 19th Century to recent years. This set of data cannot be considered as a complete survey of the historical world’s fisheries, but we believe that the result that we obtained are sufficiently robust to show that overexploitation is an important component of several cases of fishery decline. More cases can probably be analyzed in this way if it will be possible to find suitable data for the capital variable.

### 3.1 American whaling during the 19th century

**Fig. 3:**
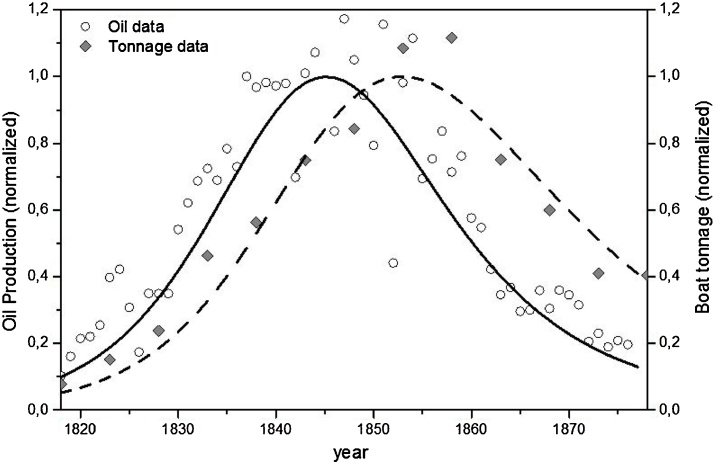
Lotka-Volterra modeling of American Whaling on the 19th century. Sperm Oil (production-prey) and the Tonnage capacity of fishing boats (capital-predator) from 1818 to 1878. Normalizing factors: oil 1.16 10^5,^ gallons, Boat tonnage 9.72 10^4^.

The first documented evidence of the overexploitation of a marine resource goes back approximately two centuries ago when the American whaling industry went through an intensive development phase due to the growing demand of whale-derived products as the whale oil, used as fuel for lamps. Whales also produced “whale bone,” a ‘stiffener for ladies’ clothing that was described as ‘the plastics of the 19th Century’(Bardi, 2007). Here, we examined the data reported in Alexander Starbuck's book about the history of the American whale fisheries from 1807 to 1876 (Starbuck, 1989). The sum of the production of oil from ‘right’ and ‘sperm’ whales, the two-main species hunted, is assumed to be proportional to the predation activity. The proxy for the predator stock is assumed to be the total tonnage of the whaling fleet. These data could be well fitted by the simplified LV model (Fig. 3), as reported in an earlier study (Bardi and Lavacchi, 2009). The definitive collapse of the American whaling industry occurred at the beginning of 20th Century, as reported by Walter Tower (Tower, 1907) and more recently by Granville Mawer (Mawer, 2000).

Data Source: Starbuck (1989). History of the American Whale Fishery. Castle. The GF for the fitted data: NMSE Oil fit: 0.76; NMSE Tonnage fit: 0.85.

### 3.2 The California sardine fishery

The Pacific sardine fishery began to operate in central California in the late 1800s and its production grew in response to the increasing demand for food during World War I (Schaefer et al., 1951). From the mid-1930s to the mid-1940s this fishery was the largest in the Northern and Southern West Pacific coasts with peak landings of over 790000 tons per year in 1936–1937 and average landings over 600000 tons per year. The fishery began to collapse a few years later and catches declined over the following decades to less than 100 tons per year in the 1970s (Radovich, 1982). A moratorium was applied to the sardine fishing from 1974 to 1986 (Wolf, 1992), resulting in a new fishery cycle that will not be examined here as we can’t define it as a “non-managed” fishery anymore.

**Fig. 4:**
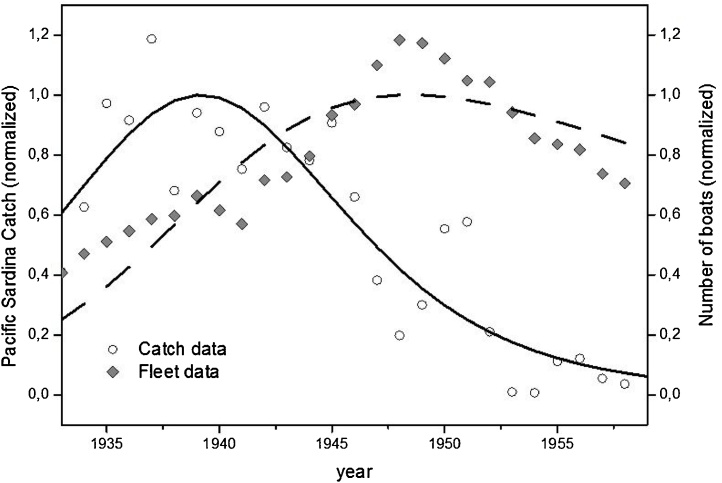
Lotka Volterra modeling of Californian Sardines Fishery. Catch (production-prey) and the number of fishing boats (capital-predator) from 1933 to 1959. Normalizing factors: catch 6.12 10^5^ Tons, the number of boats 5.21 10^3^.

In Fig. 4, we report the Californian sardine catch as production in comparison to the fishing fleet number of boats as capital, from 1933 up to 1957 and the related fitting obtained by LV modeling.

Data Source: Fish Bulletin’s, Fish Catch Statistics, California explores the Ocean website (https://library.ucsd.edu/ceo/). The GOF for the fitted data: NMSE Catch fit: 0.77; NMSE Fleet fit: 0.75.

The model shows how fishing “capital” in terms of the number of fishing boats reached the peak ten years later than production peak, leading to the fishery collapse. Again, we see that the Lotka-Volterra model provides a reasonably good fitting of the experimental data, indicating that the sardine stock was overexploited by the fishing industry.

### 3.3 Japan fishery industry

**Fig. 5:**
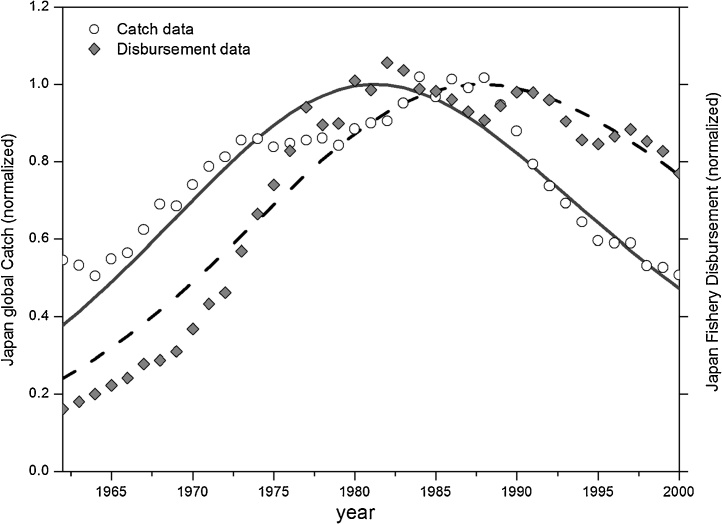
Lotka Volterra modeling of Japanese Fishery Industry. Total Catch (production-prey) and the Disbursement of Fishery (capital-predator) from 1962 to 2000. Normalizing factors: catch 1.26 10^7^ Tons, disbursement 1.35 10^8^ Yen.

The prey-predator dynamic can describe not only the exploitation of a single species; it is also able to illustrate the exploitation tendency of a whole fishing sector of a country. Here we report the evolution of the Japanese total fish catch and the National Disbursement for the fishing industry from 1962 to 2002. The catch data are expressed as the total landings by weight. The disbursement is expressed in currency and it includes expenses for the fisheries’ in terms of wages, fuel, fishing boats capital and replacement and equipment. This is a direct measurement of the capital investment in the sector. As shown in Fig. 5, the historical data show a decline in the fishery yield starting with approximately 1980. The fitted peak of the capital expenditure occurs later, although the oscillations of the data indicate an earlier peak.

Data Source: Statistics Bureau, Ministry of Internal Affairs and Communications (http://www.stat.go.jp/). The GOF for the fitted data: NMSE Catch fit: 0.81; NMSE Disbursement fit: 0.92.

Recent data from the Statistical Handbook of Japan 2015 (OECD, 2015) show that, since 2000, the Japanese fish production trend is still declining. The value of the catch is decreasing with a rate of 25% from 2000 to 2014. For the same period, the Statistical Handbook of Japan 2015 also reports the number of Enterprises and the number of workers engaged in the Fishery sector. The values of such entities, even though they are not expressed in currency, can be reasonable assumed proportional to the capital effort invested in the sector. The data show that the trend, for both, is declining: in particular, from 2000 to 2014, the number of enterprises is reduced by 39%, while the number of workers is reduced by 33%.

### 3.4 Iceland fishing industry

**Fig. 6:**
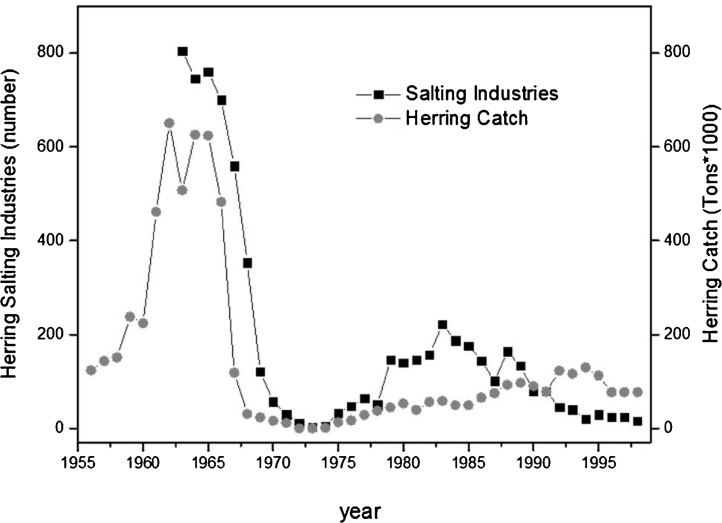
Total Icelandic Herrings catch from 1956 to 1998. Catch (in Tons*1000) in comparison to the number of Salting Herrings Industries. Data Source Statistics Iceland (http://www.statice.is/).

Iceland is another region whose economy was historically based on fishing, in particular cod and herrings. In the 1970s, the Icelandic fishing zone has been the theater of the so-called ‘cod wars’ which saw the Icelandic fleet engaged in competition against foreign fleets. By the early 1970s, the most valuable cod fish stocks had declined to very low levels of catch as a result of overfishing. In the same period, the North Atlantic Icelandic spring and summer spawning herring stock collapsed. The spring stock has not recovered yet, although the summer stock did (Jakobsson, 1980), (Jakobsson and Stefansson, 1999). The herring collapse provoked the drastic fall in the number of Herring Salting industries a few years later (Fig. 6).

**Fig. 7:**
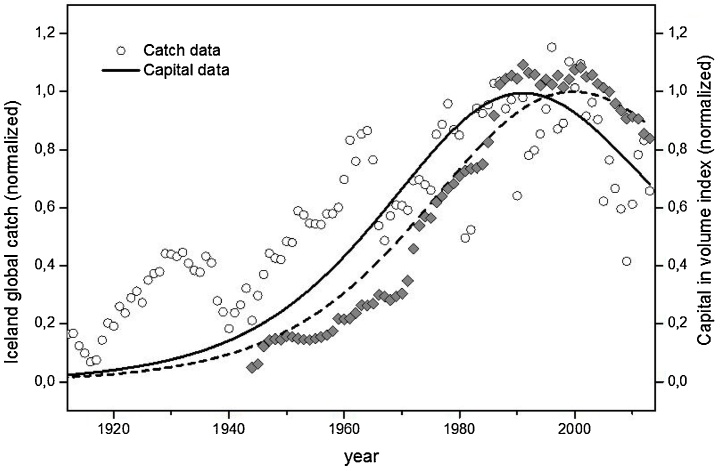
Lotka Volterra modeling of Iceland Fishery Industry. Total catch (production-prey) and Gross Fixed Capital Formation (GFCF) for Fishing expressed in Volume Index (capital-predator) from 1905 to 2014. GFCF index data are available only from 1945. Normalizing factors: catch 1.64 10^6^ Tons, Volume index at constant price 9.76 10^1^ Million ISK/index (data 1990–2014 index = 100 reference year 2005; data 1945–1990 index = 100 reference year 1990).

As a response to the depletion of such important stocks, in 1984 Iceland established an individual quota system (ITQs) for fishing vessels that allocates maximum catch on the base of past years’ vessel’s catch performance. In recent years the country has adopted a “total allowable catch” (TAC) regulation and other management measures such as area restrictions and fishing gear restrictions. Despite these measures, the curve of the total catch of the Iceland from 1905 up to 2014, shows a peak in the late 80 s (Fig. 7) followed by the capital peak, approximately ten years later, this time represented in form of economic index, the volume index at a constant price.

Data Source: Statistics for Iceland (http://www.statice.is/). The GOF for the fitted data: NMSE Catch fit: 0.61; NMSE Index fit: 0.96.

### 3.5 Other cases of overfishing

The scientific literature reports several other documents on historic fisheries collapses, such as the famous case of North Atlantic cod collapse in Newfoundland (Hutchings and Myers, 1994), although it is not always possible to detect a smooth bell-shaped curve. Nevertheless, the evidence of overexploitation is still detectable in the fact that the “capital” parameter keeps growing for a period, while the production of the fishery declines. A good example is that of the UK trawling fishery that saw a rapid decline in an intensive use of trawlers over 100 years (Thurstan et al., 2010).

**Fig. 8:**
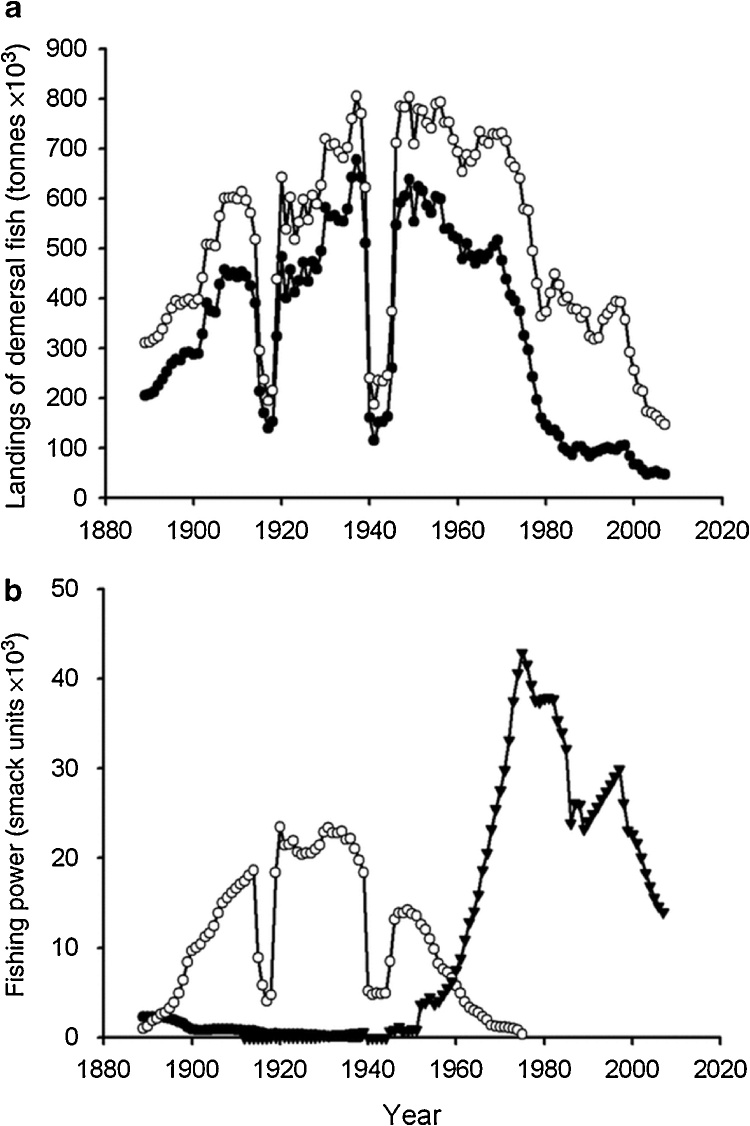
The collapse of the UK trawling fishery. By Thurstan et al., 2010. Note how the “fishing power” has continued to increase despite the decline in the catch. This is a typical feature of the model described in the present paper.

The decline occurred despite the considerable increase of the “fishing power,” something that can be likened to the parameter called here fishing capital (Fig. 8).

**Fig. 9:**
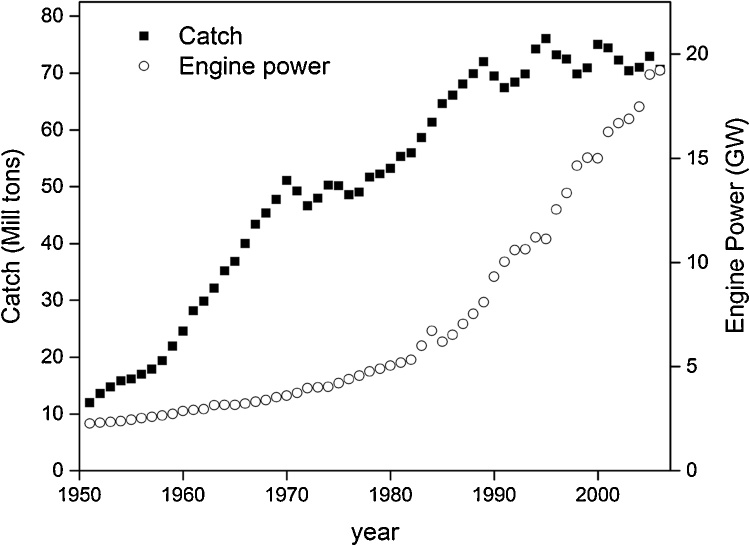
World Fish Catch. The trend in catch (Millions of Tons) per unit of effort on power use (in Giga Watt) by fishing vessels (Pauly and Zeller, 2016).

Then, the same behavior can be seen at the planetary level where the LV model appears to be able to qualitatively describe the overexploitation of marine fisheries. Statistics from FAO (FishStat) show that the world landings reached a peak in the middle of the 1990s, then started declining; a still ongoing phenomenon. Such a behavior can be correlated with the engine power of the global fishing fleet (Fig. 9) (Watson et al., 2013), that is another “proxy” variable to measure the capital invested in fishing at World level. The work of Pauly and Zeller, (2016) also shows a similar behavior. This set of data cannot be yet fitted with the LV model developed here, but it shows a behavior compatible with the model’s results.

## 4 Discussion

The problem of overfishing is much debated in the scientific community but it is often difficult to separate the effect of overexploitation from other factors involved in fishing. Sometimes, indeed, overexploitation is underestimated or even ignored in the analysis, a tradition that may have started with the early study by Starbuck (Starbuck, 1989) of the American whale fishery, where the decline of the catch was explained by such consideration as “the whales have become shy.” However, from the analysis performed here, it appears that overexploitation is, indeed, a major element in determining the behavior of fisheries.

The dynamical model we used shows that, in many cases, the fishery yield is directly linked to the magnitude of the fish stock and that fishing continues all the way to bringing the yield to nearly zero by increasing the fishing effort despite fish depletion and consistently increased the fishing effort disregarding the risk of the collapse of the fish stock. These results evidence that today, despite the necessary efforts already put in the stock preservation, as fishing policy and regulations adopted by the majority of the countries, the fish demand on the market still drives the fishing pressure where fishing remains intensively practiced worldwide, with an increasing fishing power in terms of more sophisticated and expensive fishing techniques or equipment.

In the history of fisheries, the most common measure adopted to avoid the collapse of the fish stock has been the total moratorium on fishing. In some cases, this approach has led to the stock ‘salvation’ as in the case of the Californian sardine (end of 1950), whaling (1985), the Canadian North Atlantic cod (1992), but it was a remedy, not really a solution able to prevent the related fishery industry collapse. Moreover, the time lapse necessary for the recovery of stocks has often been quite long in comparison the time-scale of the development of the modern fishing economy, that requires meeting the world demand. On this point, it is sufficient to remember that the “right whale” fishery has not yet recovered from the intensive overexploitation it suffered in the 19th Century (Baker and Clapham, 2004).

Today, in order to prevent the collapse of fisheries, many international, national, and governmental organizations establish quotas for commercial stocks, expressed in terms of “total allowable catch” (TAC). This limit is deliberated on the basis of advice from scientific advisory bodies. Despite quotas, however, there exist several historical and recent cases where stock collapses still occurred, as shown by the case of the closure of the sardine fishing season off the length of the U.S. West Coast, recommended by the Pacific Fishery Management Council (*Decision Summary Document Pacific Fishery Management Council*, 2016) in April 2016. It was closed mid-season in 2015 due to low stocks, but it has since fallen further. Federal rules mandate that the harvest must be closed if adult stocks fall below 150,000 tons, and the government estimates that there are now less than 65,000 tons.

The innovative approach of the present study is that it describes the quantitative correspondence between a biological resource and a capital by which the dynamic of overfishing can simply be represented. We are not just fitting data, but interpreting them with a model that specifies the dynamic interactions within its internal elements, so that obtaining a good fit indicates that the corresponding elements in the real world do behave according to the assumptions made in the model. We believe that our results are important in highlighting the fundamental role that overexploitation is playing in the cycle of modern fisheries, confirming the interpretation, for instance, by Daniel Pauly (Pauly, 2009) of the global fishing trends. Overexploitation needs to be understood if it is to be avoided by means of appropriate measures such as quotas, restrictions, or sustaining fisheries toward the transition to a circular economy. Our model could also be used to provide elements useful to establish fishing quotas, but this will be the objective of further studies.

## 5 Conclusion

The scope of the present study was to evidence how the abstract concept of “overexploitation” can be detected and analyzed by a dynamic model derived from the well-known Lotka-Volterra, “prey/predator” model. The innovative approach of this model is that it is a quantitative application of system dynamics that emphasizes the role of depletion and the feedback relationships between the various parameters of the models. The history of fisheries provides us with several cases where the behavior of the fishery can be described by a model that assumes that the economic yield of the fishery is directly proportional to the investments in the field. With the reduction of the yield generated by overexploitation, the result is a general decline in the landings, followed by a decline of the effort of the fishing industry that comes after a certain time lapse. The resulting curves show a typical “bell-shaped” behavior that can be fitted with the model described here that appears to be able to can capture the behavior of a number of cases where the fishing industry operates in an open market framework. Of course, from the number of cases examined, we cannot claim that the model describes all the sectors of the fishing industry, nor that overexploitation is the only forcing that affects the system. Nevertheless, we believe that it catches some important factors that drive the industry to overexploit the fish stocks. This result shows the importance of the problem of overexploitation in the general issue of the management of the “global commons” that include much more stocks of resources other than fisheries.

## References

[bib0005] Bailey J. (2016). Adventures in cross-disciplinary studies: grand strategy and fisheries management. Mar. Policy.

[bib0010] Baker C.S., Clapham P.J. (2004). Modelling the past and future of whales and whaling. Trends Ecol. Evol..

[bib0015] Bardi U., Lavacchi A. (2009). A simple interpretation of Hubbert’s model of resource exploitation. Energies.

[bib0020] Bardi U., Lavacchi A., Yaxley L. (2011). Modelling EROEI and net energy in the exploitation of non renewable resources. Ecol. Modell..

[bib0025] Bardi U. (2007). Energy prices and resource depletion: lessons from the case of whaling in the nineteenth century. Energy Sources Part B Econ. Planning. Policy.

[bib0030] D’Ancona U. (1942). Una lotta per l’esistenza, Enaudi Editore.

[bib0035] Decision Summary Document Pacific Fishery Management Council 2016 at http://www.pcouncil.org/resources/archives/council-meeting-decisions/.

[bib0040] Forrester J. (1989). The beginning of system dynamics Banquet Talk at the international meeting of the System Dynamics Society. McKinsey Quarterly.

[bib0045] Gause G.F. (1932). Experimental studies on the struggle for existence: I: mixed population of two species of yeast. J. Exp. Biol..

[bib0050] Gordon H.S. (1954). The economic theory of a common-property resource: the fishery. J. Polit. Econ..

[bib0055] Hall C.A.S. (1988). An assessment of several of the historically most influential theoretical models used in ecology and of the data provided in their support. Ecol. Modell..

[bib0060] Hardin G. (1968). The tragedy of the commons. Science (80-.).

[bib0065] Hutchings J.A., Myers R.A. (1994). What can be learned from the collapse of a renewable resource? Atlantic cod, Gadus morhua, of Newfoundland and Labrador. Can. J. Fish..

[bib0070] Jakobsson J., Stefansson G. (1999). Management of summer-spawning herring off Iceland. ICES J. Mar. Sci..

[bib0075] Jakobsson J. (1980). Exploitation of the Icelandic Spring and Summer Spawning Herring in Relation to Fisheries Management 1947–1980.

[bib0080] Lotka J. Alfred (1925). Elements of Physical Biology.

[bib0085] Lotze H.K., Worm B. (2009). Historical baselines for large marine animals. Trends Ecol. Evol..

[bib0090] Mawer G.A. (2000). Ahab’s Trade: The Saga of South Seas Whaling.

[bib0095] OECD (2015). Japan 2015. OECD Economic Survey.

[bib0100] Pauly D., Zeller D. (2016). Catch reconstructions reveal that global marine fisheries catches are higher than reported and declining. Nat. Commun..

[bib0105] Pauly D. (2009). Aquacalypse Now [WWW Document].

[bib0110] Perry A.L., Low P.J., Ellis J.R., Reynolds J.D. (2005). Climate change and distribution shifts in marine fishes. Science.

[bib0115] Polis G.A., Strong D.R. (1996). Food web complexity and community dynamics author gary A. polis and donald R.. strong source. Am. Nat..

[bib0120] Radovich J. (1982). The collapse of the California sardine fishery what have we learned?. CalCOFI Reports XXIII.

[bib0125] Roopnarine P. (2013). Ecology and the Tragedy of the Commons. Sustainability.

[bib0130] Schaefer M.B., Sette O.E., Marr J.C. (1951). Growth of Pacific Coast Pilchard Fishery to 1942 U.S.

[bib0135] Schaefer M.B. (1957). Some considerations of population dynamics and economics in relation to the management of the commercial marine fisheries. J. Fish. Res. Board Can..

[bib0140] Schoener T.W. (1976). Alternatives to lotka-Volterra competition: models of intermediate complexity. Theor. Popul. Biol..

[bib0145] Starbuck A. (1989). History of the American Whale Fishery.

[bib0150] Thurstan R.H., Brockington S., Roberts C.M. (2010). The effects of 118 years of industrial fishing on UK bottom trawl fisheries. Nat. Commun..

[bib0155] Tower W.S. (1907). A History of the American Whale Fishery, Publications of the University of Pennsylvania. Series in Political Economy and Public Law.

[bib0160] Volterra V. (1928). Variations and fluctuations of the number of individuals in animal species living together. J. du Cons. Perm. Int. pour l’ Explor. la Mer.

[bib0165] Volterra V., Chapman R.N. (1931). Variations and fluctuations of the number of individuals in animal species living together. Animal Ecology..

[bib0170] Hilborn R., Walters C.J. (1992). Quantitative Fisheries Stock Assessment: Choice, dynamics and Uncertainty.

[bib0175] Watson R.A., Cheung W.W.L., Anticamara J.A., Sumaila R.U., Zeller D., Pauly D. (2013). Global marine yield halved as fishing intensity redoubles. Fish Fish..

[bib0180] Wolf P. (1992). Recovery of the pacific sardine and the california sardine fishery. CalCOFI Rep..

[bib0185] Worm B., Hilborn R., Baum J.K., Branch T.A., Collie J.S., Costello C., Fogarty M.J., Fulton E.A., Hutchings J.A., Jennings S., Jensen O.P., Lotze H.K., Mace P.M., McClanahan T.R., Minto C., Palumbi S.R., Parma A.M., Ricard D., Rosenberg A.A., Watson R., Zeller D. (2009). Rebuilding global fisheries. Science.

